# Bioprospecting CAZymes repertoire of *Aspergillus fumigatus* for eco-friendly value-added transformations of agro-forest biomass

**DOI:** 10.1186/s13068-023-02453-6

**Published:** 2024-01-03

**Authors:** Namrata Joshi, Jasneet Grewal, Lukasz Drewniak, Kumar Pranaw

**Affiliations:** Department of Environmental Microbiology and Biotechnology, Institute of Microbiology, Faculty of Biology, University of Warsaw, Miecznikowa 1, 02-096 Warsaw, Poland

**Keywords:** Lignocellulosic waste, Pine sawdust, Solid-state fermentation, Response surface methodology, Secretome analysis, Enzymatic hydrolysis

## Abstract

**Background:**

Valorizing waste residues is crucial to reaching sustainable development goals and shifting from a linear fossil-based economy to a circular economy. Fungal cell factories, due to their versatility and robustness, are instrumental in driving the bio-transformation of waste residues. The present work isolated a potent strain, i.e., *Aspergillus fumigatus* (ZS_AF), from an ancient Złoty Stok gold mine, which showcased distinctive capabilities for efficient hydrolytic enzyme production from lignocellulosic wastes.

**Results:**

The present study optimized hydrolytic enzyme production (cellulases, xylanases, and β-glucosidases) from pine sawdust (PSD) via solid-state fermentation using *Aspergillus fumigatus* (ZS_AF). The optimization, using response surface methodology (RSM), produced a twofold increase with maximal yields of 119.41 IU/gds for CMCase, 1232.23 IU/gds for xylanase, 63.19 IU/gds for β-glucosidase, and 31.08 IU/gds for FPase. The secretome profiling validated the pivotal role of carbohydrate-active enzymes (CAZymes) and auxiliary enzymes in biomass valorization. A total of 77% of carbohydrate-active enzymes (CAZymes) were constituted by glycoside hydrolases (66%), carbohydrate esterases (9%), auxiliary activities (3%), and polysaccharide lyases (3%). The saccharification of pretreated wheat straw and PSD generated high reducing sugar yields of 675.36 mg/g and 410.15 mg/g, respectively.

**Conclusion:**

These findings highlight the significance of an efficient, synergistic, and cost-effective arsenal of fungal enzymes for lignocellulosic waste valorization and their potential to contribute to waste-to-wealth creation through solid-waste management. The utilization of *Aspergillus fumigatus* (ZS_AF) from an unconventional origin and optimization strategies embodies an innovative approach that holds the potential to propel current waste valorization methods forward, directing the paradigm toward improved efficiency and sustainability.

**Supplementary Information:**

The online version contains supplementary material available at 10.1186/s13068-023-02453-6.

## Background

To effectively address the detrimental impacts of fossil fuels, it is widely acknowledged that sustainable development necessitates a shift from the existing linear economic system to a circular model. The primary objective of the circular model is to create a sustainable and regenerative economic system by reducing dependency on finite resources and promoting the use of renewable biomass feedstocks [1]. With a worldwide annual production of 1 × 10^10^ metric tons, lignocellulosic biomass has piqued the interest of researchers, accounting for approximately half of the biosphere's total biomass reserves [2]. Due to their abundance, low cost, and sustainable nature, agricultural and forest residues constitute a compelling supply of lignocellulosic biomass feedstock. Lignocellulosic biomass mainly comprises cellulose, hemicellulose, and lignin, with minor amounts of extractives and inorganic components varying according to the wood, grass, or sedge type.

Among the forest residues, sawdust from pine [3], poplar [4], birch [5], eucalyptus [6], etc., cultivated worldwide, represents a major low-cost by-product abundantly available from wood transformation processes but not effectively utilized due to their structural heterogeneity and recalcitrance [7]. According to the European Organization of Sawmill Industries (EOS), sawdust production averaged 13.4 million m^3^ between 2020 and 2022. Depending on market demand, sawdust can be supplied to the particle board industry, utilized as a renewable fuel to produce bio-energy (e.g., pellets manufacture), or subjected to various procedures for the valorization of its cellulose, hemicellulose, and lignin fractions [8, 9]. However, in practice, the substantial sawdust volumes are improperly disposed of, posing significant environmental risks to air, water, and soil quality. Burning leftover sawdust outdoors pollutes the air and has unfavorable consequences for the land, such as raising its acidity levels [10]. Thus, implementing measures to properly manage leftover sawdust can alleviate environmental problems connected with its mishandling and provide economic benefits [11].

In this context, deploying sawdust as a solid substrate for cultivating microorganisms via solid-state fermentation (SSF) can be a simple, low-investment approach for producing value-added products like hydrolytic enzymes with varied biorefinery applications. SSF has advantages over submerged fermentation (SmF), including lower operational expenses, little chance of contamination, easy enzyme recovery, and the production of enzymes with higher efficiency, specific activity and easy recovery [12, 13]. However, unlike conventional agro-residues such as wheat, rice straw, corn stover, and sugarcane bagasse used as attractive fermentable materials, the reports on the usage of sawdust as a supporting matrix for biotransformation are scanty. Furthermore, the nutritional profile of pine sawdust (PSD) due to the presence of lignin-derived phenolic, resinous compounds makes it less favorable for the growth of microbes [14]. Nevertheless, fungi, with their well-known adaptability and resilience to harsh environmental conditions and a diverse arsenal of extracellular enzymes, can play a critical role in depolymerizing these complex, underutilized substrates into various valuable metabolites through SSF.

The goal of the current study was to isolate lignocellulolytic fungi from the wooden remnant structures, the scaffolding of the mine’s tunnels and corridors, taking into account that extreme environmental niches like the abandoned Złoty Stok gold mine (southwestern Poland) can be a vital source of robust adaptive organisms [15, 16]. The isolated fungi were evaluated for their biotransformation potential by pine sawdust (PSD) valorization to produce economic hydrolytic enzymes (cellulases, xylanases and β-glucosidases). To further gain insight into the lignocellulose deconstruction potential of the selected isolate, i.e., *Aspergillus fumigatus* (ZS_AF), statistical optimization of SSF critical process parameters and secretome analysis was conducted using LC–MS/MS. The CAZymes (carbohydrate-active enzymes) repertoire of *A. fumigatus* grown on PSD is scanty. Although the secretome profile varies with environmental circumstances, the discoveries will open up new avenues for waste management regarding economic value generation. Lastly, cheaply produced hydrolytic enzymes used wheat straw (WS) and PSD as model substrates for saccharification. The released fermentable sugars can generate a plethora of high-value acids, materials, platform chemicals, etc., to meet commercial demands, while the residual solid substrates can be added back to the soil to act as a carbon sink.

## Results and discussion

### Molecular identification of the isolated cellulolytic fungus

On the 5th day of incubation, *A. fumigatus* from an ancient gold mine with the highest cellulolytic activity, i.e., CMCase and FPase with 6.33 and 1.0 IU/ml, respectively, was chosen for further study. The fungus was identified based on differences in the ITS region sequencing. BLAST analysis was used to determine the similarity of the ZS_AF sequence to that of *Aspergillus fumigatus*, and evolutionary analysis was performed using the MEGA 11 program (as shown in Additional file 1: Fig. S1a). Furthermore, the sequence of *A. fumigatus* was submitted to the NCBI GenBank database under the accession number OM258166.

### Statistical optimization of hydrolytic enzyme production

**Fig. 1:**
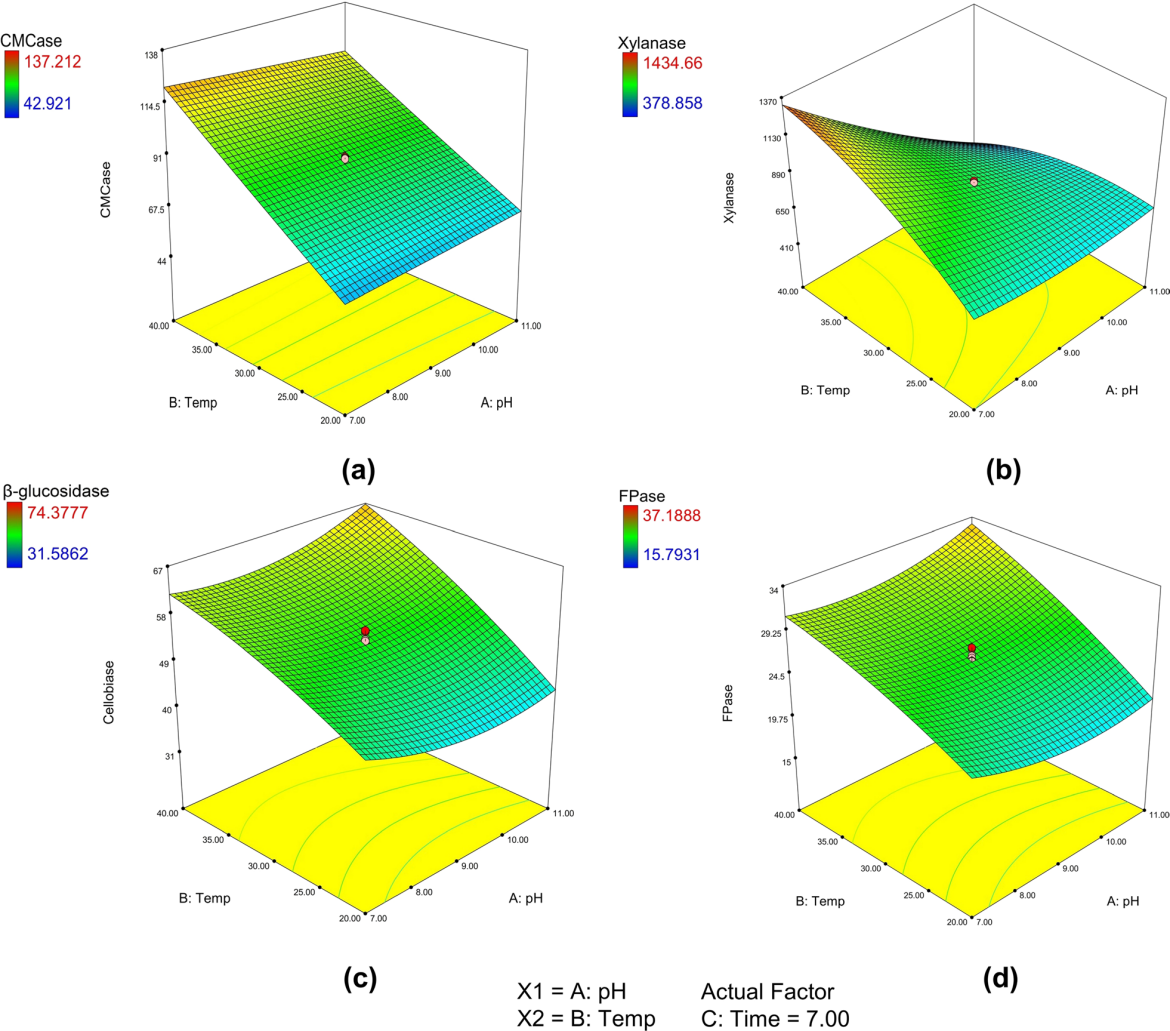
The response surface contour plot and 3D response plot for *A. fumigatus* enzymes **a** CMCase, **b** xylanase, **c** β-glucosidase, and **d** FPase as a function of pH (**A**), temperature (**B**), and time (**C**)

The employment of a regression model, particularly the response surface methodology (RSM), facilitated the exploration and maximization of enzyme production from the fungus *Aspergillus fumigatus* (ZS_AF). Based on the constant one variable at a time (COVT) approach, the enzyme yields of 60.7 IU/gds CMCase, 668.14 IU/gds xylanase, 56.4 IU/gds β-glucosidase, and 11.2 IU/gds FPase were obtained in SSF under optimized conditions of 5 g PSD as a substrate, 5 days of incubation, pH 7.0, and temperature 30 ℃. For instance, Matrawy et al. [17], demonstrated a similar approach, employing RSM to optimize enzyme production. This work illustrated the practical application and validity of the regression model in enhancing enzyme yields, aligning with the methodology employed in our study. The SEM images supported the luxuriant proliferation of *A. fumigatus* over PSD during SSF (Additional file 1: Fig. S1b, c). For the maximal yields of all four enzymatic activities and the interactive effects of physical factors such as production media pH, temperature, and incubation time on enzyme production, RSM was employed using a rotatable central composite design (RCCD). Moreover, a statistical model for the effects of these factors was derived from the RCCD experiments, where the observed and predicted responses for CMCase, xylanase, β-glucosidase, and FPase were in good agreement (Table 1). Three-dimensional response surface plots (Fig. 1) were created using the models to show the individual and interacting effects of the process factors. The ANOVA results of the quadratic response-surface model fitting for CMCase, xylanase, β-glucosidase, and FPase are presented in Additional file 1: Tables S1, S2, S3, and S4, respectively. The analysis of variance revealed that selected parameters (i.e., pH, temperature, and time) were highly significant in the production of hydrolytic enzymes. The F-value compares the lack of fit variance to pure error variance. A significant model has a high F-value. Thus, the attained respective values for CMCase (11390.12), xylanase (2082.73), β-glucosidase (52.29), and FPase (47.27) indicated that the model was significant for the production of all four enzymatic activities.

**Table 1:** RCCD experimental design developed using Design-Expert version 7.0.0 software

Run no.	Factor A pH	Factor B temperature	Factor C time	Different responses							
				CMCase (IU/gds)		Xylanase (IU/gds)		β-Glucosidase (IU/gds)		FPase (IU/gds)	
				Observed	Predicted	Observed	Predicted	Observed	Predicted	Observed	Predicted
1	7	20	5	42.92	42.50	681.76	688.47	47.48	22.99	23.74	22.99
2	11	20	5	50.08	50.11	651.35	658.15	41.70	20.22	20.85	20.22
3	7	40	5	126.31	126.34	1434.66	1429.54	69.35	34.47	34.68	34.47
4	11	40	5	121.90	121.75	548.64	555.37	74.34	36.06	37.19	36.06
5	7	20	9	80.40	80.43	853.34	847.35	54.87	27.45	27.43	27.45
6	11	20	9	82.26	82.11	749.18	755.04	53.95	26.35	27.25	26.35
7	7	40	9	118.76	118.60	1315.1	1309.05	61.37	30.06	30.54	30.06
8	11	40	9	107.77	108.06	378.86	372.89	67.97	33.30	33.66	33.30
9	5.64	30	7	91.15	91.40	1361.96	1368.54	61.54	30.95	30.64	30.95
10	12.36	30	7	89.01	88.94	563.46	555.83	60.28	31.35	30.09	31.35
11	9	13.18	7	44.56	44.81	419.06	411.47	31.57	16.61	15.79	16.61
12	9	46.82	7	137.21	137.14	706.74	713.28	62.93	32.11	31.34	32.11
13	9	30	3.64	81.91	82.14	976.24	967.61	57.77	29.97	28.89	29.97
14	9	30	10.36	102.60	102.54	940.19	947.77	61.83	31.41	30.92	31.41
15	9	30	7	89.90	89.95	833.21	834.59	54.77	26.62	27.39	26.62
16	9	30	7	89.90	89.95	846.26	834.59	55.00	26.62	27.50	26.62
17	9	30	7	90.34	89.95	831.34	834.59	53.23	26.62	26.61	26.62
18	9	30	7	90.36	89.95	832.59	834.59	52.77	26.62	26.38	26.62
19	9	30	7	89.77	89.95	833.21	834.59	52.14	26.62	26.07	26.62
20	9	30	7	89.44	89.95	830.72	834.59	52.96	26.62	26.01	26.62

The Fisher F test also indicated significance, with a probability value well below the threshold (*P* > F—0.0001). There was little probability that noise could have produced a “Model *F*-value” this significant (0.01%), which means the model could explain 99.99% of the total variation, including the sample variation, but not the final 0.01%. Model terms with “Prob > *F*” values less than 0.0500 were considered significant. The signal-to-noise ratio is measured by “Adequate Precision”. It contrasts the average prediction error with the range of the anticipated values at the design points. A ratio of at least 4 is preferred, and the signal-to-noise ratios for CMCase, xylanase, β-glucosidase and FPase in the current investigation were 392.084, 166.179, 29.068, and 27.653, respectively, indicating a sufficient signal. The regression equation generated a coefficient of determination (R^2^) of 0.9999 for CMCase, 0.9995 for xylanase, 0.9792 for β-glucosidase, and 0.9770 for FPase. The findings indicated that the quadratic model was remarkably significant and could account for nearly 95% of the variance in enzyme production.

Additionally, Additional file 1: Fig. S2 shows normal probability plots (i.e., a-d) for enzyme production of CMCase, xylanase, β-glucosidase, and FPase, respectively. The normal probability plot of residuals is a crucial diagnostic tool for identifying and clarifying any systematic deviations from the assumptions that errors are independently distributed with normal distribution and that the variances of errors are homogeneous. The average (percentage) probability plot of “Studentized” residuals suggests hardly any breach in the assumptions underlying the analyses.

After RCCD analysis, the optimal physical conditions for all three factors were determined to be pH 7.0, temperature 40 ℃, and incubation period of 5 days. The model anticipated that under the aforementioned optimum conditions, the enzyme yield for CMCase, xylanase, β-glucosidase, and FPase would reach a maximum of 126.34 IU/gds, 1429.53 IU/gds, 68.82 IU/gds, and 34.47 IU/gds, respectively. Consequently, in validation experiments conducted at the optimized values of the test variables specified by the model, the maximum enzyme production for CMCase, xylanase, β-glucosidase, and FPase was measured to be 119.41 IU/gds, 1232.23 IU/gds, 63.19 IU/gds, and 31.08 IU/gds, respectively. These outcomes were consistent with the predictions of the model. Hereafter, successful optimization using RSM resulted in an approximately twofold increase in the production of hydrolytic enzymes from the initial amount. In another study, Azzouz et al. [18] demonstrated significant enhancements through the OFAT and RSM methods, achieving maximum values of 4008.25 ± 3.73 U/gds and 5427.51 ± 4.4 U/gds, respectively. These values were notably higher than the initial conditions, which yielded 1899.02 ± 1.6 U/gds. Similarly, Kumar et al. [19] resulted in a 3.35-fold improvement in FPase activities after optimizing the medium components and concentrations using the RSM approach as compared with the un-optimized conditions un-optimized conditions. To the best of the authors' knowledge, the titres of hydrolytic enzymes obtained using PSD as a substrate are the highest ones reported to date. Furthermore, these yields are also equivalent to those reported from other popular and conventional agro-forest biomass, as listed in Table 2. These findings offer novel opportunities to valorize an underutilized but readily available waste resource.

**Table 2:** Comparative analysis of cellulolytic and xylanolytic enzymes produced by *Aspergillus* sp. growing on different agro-forest waste

Substrate	CMCase activity (IU/gds)	Xylanase activity (IU/gds)	β-Glucosidase activity (IU/gds)	FPase activity (IU/gds)	Reference
Rice straw	235	180	190	12.5	[48]
Brewery spent grain	3.24	2279.9	-	-	[49]
Biomass sorghum	41.47	300.07	63.61	-	[50]
Sugarcane bagasse	4.20	-	-	0.64	[51]
Wheat bran	20.5	-	87.6	7.8	[52]
Pine sawdust	119.41	1232.23	63.19	31.08	Present study

### Secretome analysis of degradative proteins released by *A. fumigatus* cultured on PSD

**Fig. 2:**
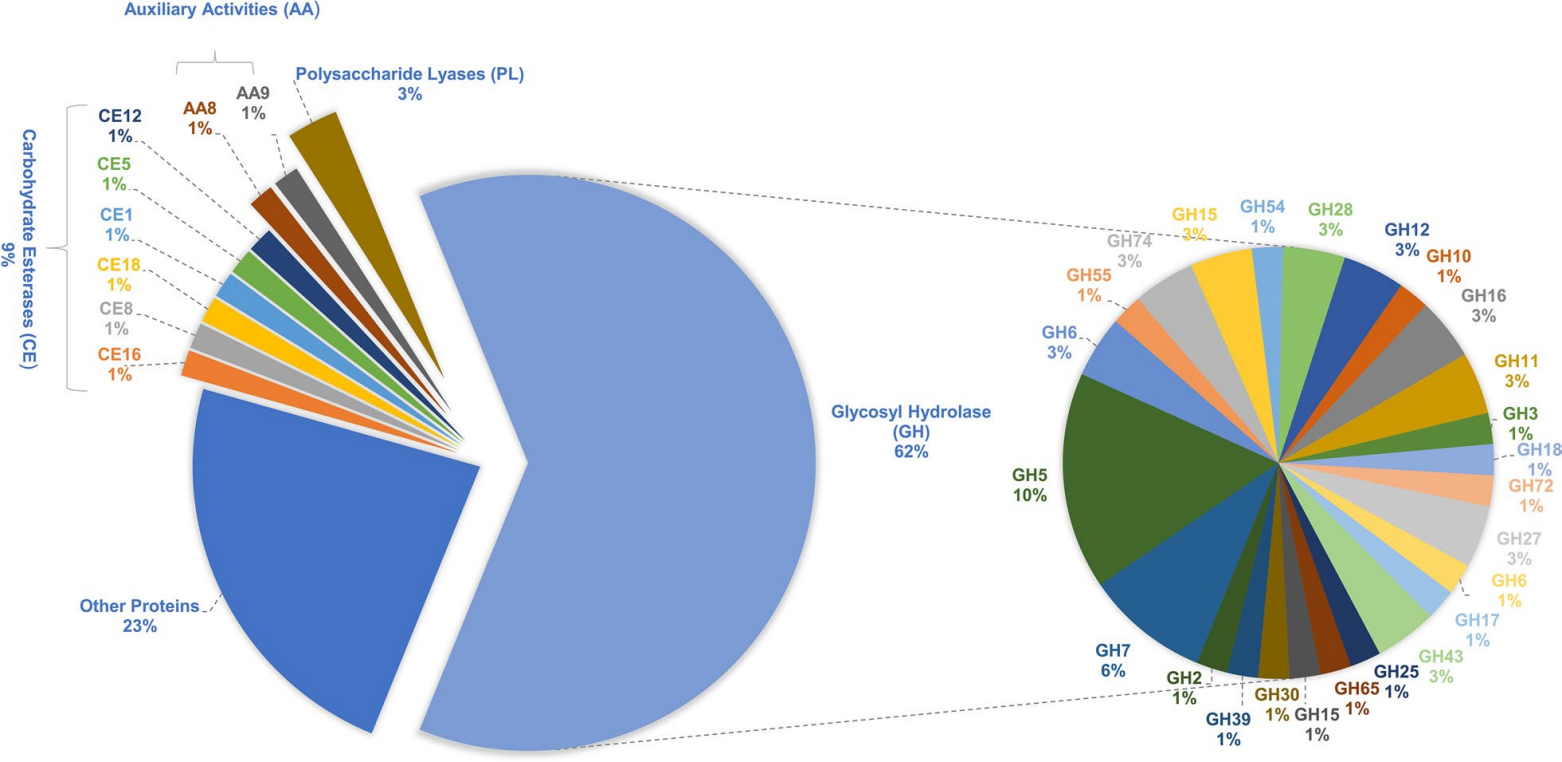
Compositional study of total proteins found in the secretome of *A. fumigatus* identified during SSF with PSD. Percentage (%) of differentially expressed CAZymes include glycoside hydrolases (GH), auxiliary activities-related enzymes (AA), carbohydrate esterases (CE), polysaccharide lyases (PL), and other non-CAZYmes

To complement the obtained enzymatic titres and elucidate the holistic enzymatic repertoire employed during solid substrate degradation, secretome analysis using LC–MS/MS was conducted. This integrated approach not only identifies key CAZymes but also sheds light on their coordinated action, enhancing valorization prospects by *A. fumigatus*. By employing PSD as a substrate, extracellular protein secretion from *A. fumigatus* during SSF was carried out. The study found 69 proteins, 77% of which were carbohydrate-active enzymes (CAZymes) (http://www.cazy.org), with 62% glycoside hydrolases (GH), 9% carbohydrate esterases (CE), 3% auxiliary activities (AA), and 3% polysaccharide lyases (PSL) (as illustrated in Fig. 2). The remaining 23% comprised hypothetical proteins, oxidoreductase, lipase, and chitinase. The study also revealed that the molecular weights of the identified proteins ranged from 12.9 to 117.2 kDa, with most of the proteins being acidic, as indicated by their isoelectric points (pI) ranging from 4 to 7. This finding was consistent with previous studies by [20, 21], who reported similar molecular weight and pI observations. As glycoside hydrolases (GH) break down lignocellulosic biomass, endoglucanases (EGs) primarily act on the amorphous region of glycosidic linkages through hydrolysis. Cellobiohydrolases (CBHs) release cellobiase by acting on the reducing and non-reducing ends of chains, while β-glucosidases liberate glucose as the end product [22]. The secretome analysis conducted in this study revealed that among all GH families, GH5 (10%), GH12 (3%), and GH16 (3%) were the most abundant, dominated by endoglucanases, followed by GH7 (6%) and GH6 (3%) which included cellobiohydrolases. In addition to cellulose-degrading enzymes, endoxylanases and xylosidases from families such as GH11 (3%), GH43 (3%), and GH10 (1%) were also observed. Adav et al. [23] observed a comparable dominance of endoglucanases and cellobiohydrolases from the GH5 and GH7 families in the *A. fumigatus* secretome. The remaining CAZymes families are involved in the degradation of other polysaccharides, such as pectin, classified under GH28, CE8, CE12, and PL1 families. The study also detected the presence of the AA9 family, including lytic polysaccharide monooxygenases (LPMOs), and various carbohydrate-binding module (CBM) families, such as CBM1 (primarily found in fungi), CBM20, CBM42, CBM19, and CBM43. The finding of secretome emphasizes the range of CAZymes produced by *A. fumigatus* during solid-state fermentation of PSD and the ability of *Aspergillus* to valorize non-conventional recalcitrant substrate.

### Structural analysis and enhancement of hydrolysis efficiency by *Aspergillus*-derived enzyme

**Fig. 3:**
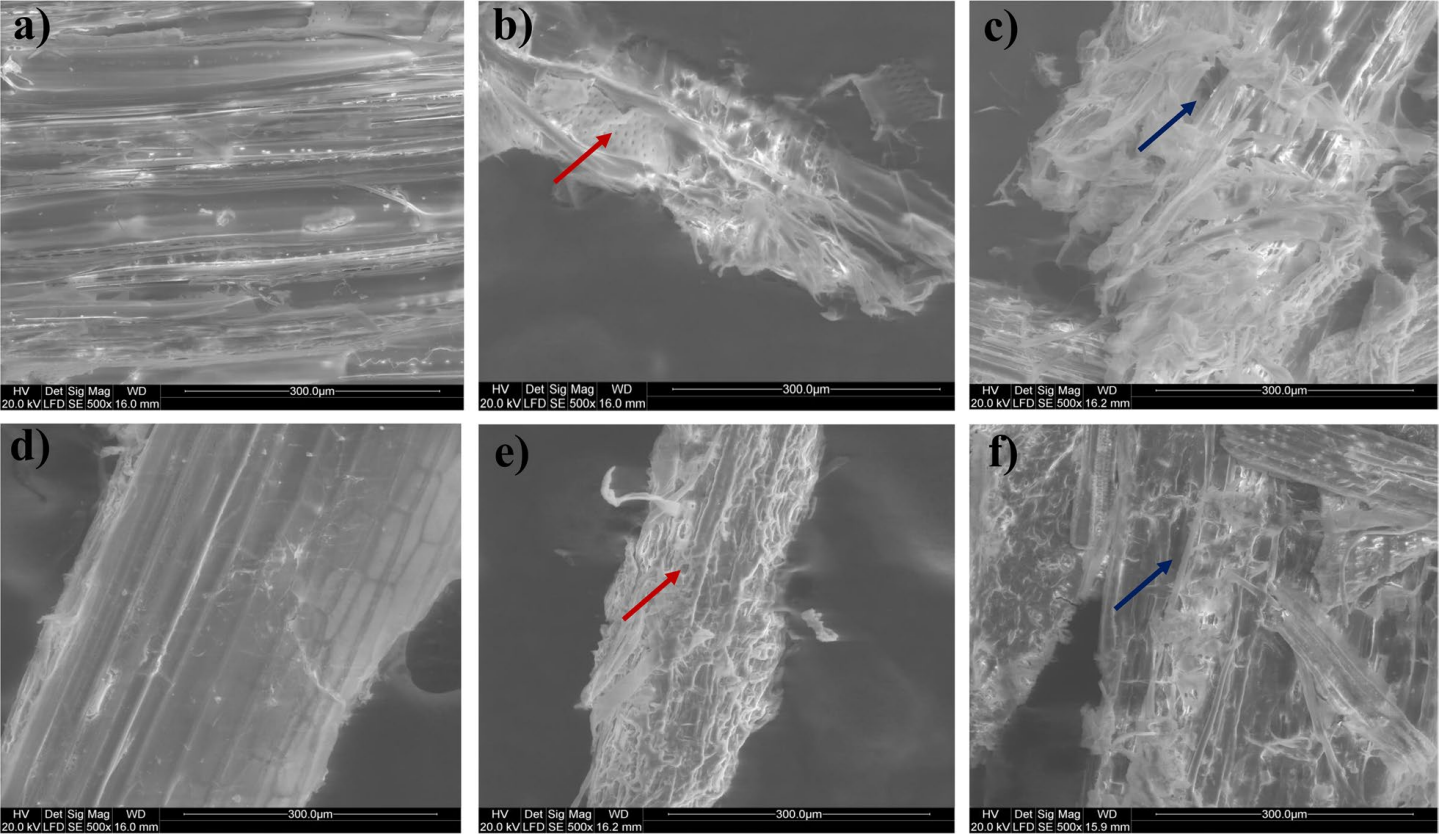
SEM detection of pine sawdust **a** untreated; **b** alkali-pretreated; **c** hydrolyzed with *A. fumigatus*-derived enzyme and wheat straw **d** untreated; **e** alkali-pretreated; **f** hydrolyzed wheat straw

The morphological structure of both substrates, i.e., PSD and WS, were examined by SEM (Fig. 3). The surface of raw PSD and WS (Fig. 3a, d) exhibited stiff, meticulously ordered fibrils, whereas, after pretreatment, their structure tends to be less ordered and leads to the formation of pores or gaps (a red arrow in Fig. 3b, e) facilitating increased availability of cellulose surface area for enzyme attack. Similarly, further disrupted fibers showed more fractures after hydrolysis (blue arrow in Fig. 3c, f). Likewise, structural disruption has also been observed in many studies with various pretreatment techniques [24, 25]. The effect of alkali pretreatment in both substrates was further supported by FTIR spectra, which showed a diminution in the absorption peak at 3437 cm^-1^ as a result of the stretching of the O-H bond of the phenol group of cellulose and lignin in comparison to the untreated substrate (Additional file 1: Fig. S3a, b). Similarly, alkaline pretreatment resulted in a disturbance in another peak at 1742 cm^-1^, representing the disruption of C=O stretching in the acetyl ester and carbonyl aldehyde units of hemicellulose and lignin, indicating the successful breakdown of hemicellulose and lignin from both substrates. Another shift in the peak at 1375 cm^-1^, corresponding to C-H deformation in cellulose and hemicellulose, was noticed, resulting in a declining trend and the peak at 1236 cm^-1^, complementary to disruption of C-O stretching in lignin fraction following processing [26, 27].

Furthermore, XRD was utilized to analyze the crystallinity of both PSD and WS raw substrates and treated with a pretreatment approach and subsequent hydrolysis, as shown in Additional file 1: Fig. S4a, b. The XRD pattern showed distinctive peaks at 2 values of 18.5° and 22.5°, corresponding to the (101) and (002) lattice planes of crystalline cellulose type I, respectively [28]. The alkali-pretreated PSD and WS showed a considerable rise in crystallinity index (CrI) of 73.21% and 68.9% compared to the untreated PSD and WS, which had a CrI of 44.2 and 58%, respectively. This rise in CrI was attributable to the pretreatment method, which increased the cellulose content by removing lignin and hemicellulose. A similar increase in CrI after pretreatment was also observed in earlier studies [4, 25]. Furthermore, after the alkaline pretreatment procedures, 4% decrease in CrI following hydrolysis in both substrates was observed due to more extensive structural disruption.

**Fig. 4:**
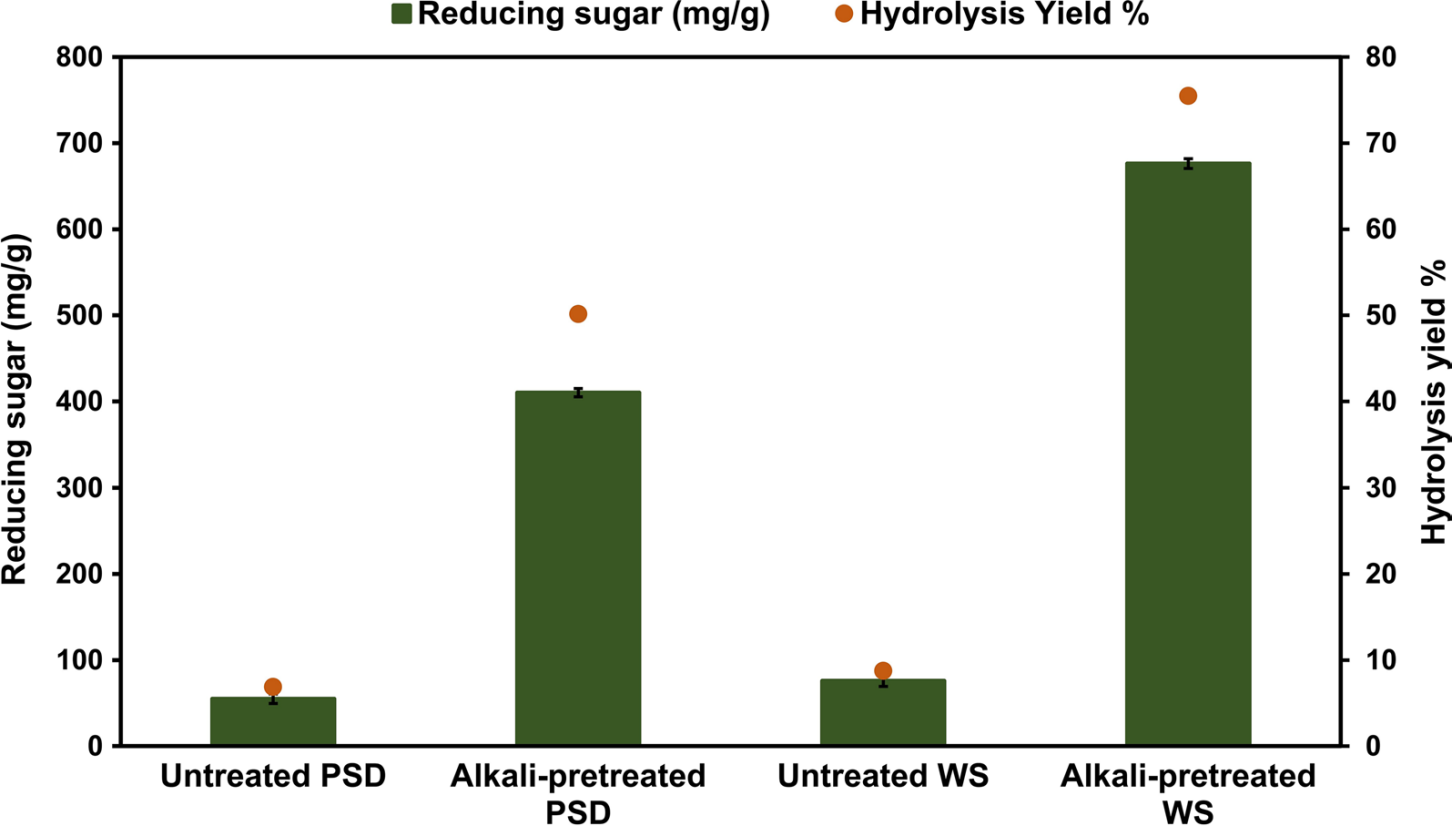
Saccharification potential of *A. fumigatus* on untreated and alkali-pretreated WS and PSD

Following the PSD and WS structural analysis, the enzymatic hydrolysis was investigated using an *A. fumigatus*-derived enzyme during SSF with PSD. After 72 h of hydrolysis, alkaline pretreated WS with 30 FPU/g cellulase loading yielded 675.26 mg/g reducing sugar. Likewise, using PSD as a substrate for saccharification resulted in a sugar yield of 410.15 mg/g using 150 FPU/g of cellulase loading (Fig. 4). As compared to untreated substrates, the hydrolysis yield of alkaline pretreated PSD and WS was enhanced by 50.15% and 75.5%, respectively. Due to the higher crystalline nature of PSD compared to WS, a higher cellulase loading was required. Liang et al. [4] and Kruyeniski et al. [29] discovered a ~55% and ~25% hydrolysis yield for two types of sawdust, popular and pine sawdust. Similarly, Jin et al. [7] found that pine and catalpa sawdust yielded 44.59 and 39.76 mg/g of reducing sugar yield, respectively. Notably, utilizing pine sawdust with its endogenously produced enzymes led to a higher reducing sugar yield while avoiding costly purification procedures. In contrast to earlier investigations, Table 3 shows that utilization of WS during saccharification resulted in significantly more reducing sugar than other reports. Zabihi et al. [30] and Ilanidis et al. [31] achieved 480 kg/ton and 336.41 g/kg of reducing sugar using commercial cellulase (ASA Spezialenzyme GmbH, Germany and Cellic CTec2) of 15 FPU and 100 CMCase, respectively. Additionally, reducing sugar generation using hydrolyze derived from fungi such as fungus consortium release reducing sugar of 402.38 mg/g [32]; *Sporotrichum thermophile* 281 mg/g [33]; *Phoma exigua* 177.2 mg/g [34] of wheat straw. The outcomes of this study were also compared to those of other lignocellulosic biomass sources. Catalpa sawdust produced 136.44 mg/g of reducing sugar [35], whereas raw wheat straw and rice straw yielded 130.24 mg/g and 125.36 mg/g of reducing sugar, respectively [36]. Using grass clippings as a substrate, on the other hand, provided a significantly more significant amount of reducing sugar at 229.42 mg/g [7]. It addresses the problems associated with waste management and the negative impacts that waste burning and accumulation cause on the environment. Addressing these difficulties could improve the economic feasibility of biorefinery processes.

**Table 3:** Yield of reducing sugars obtained from enzymatic hydrolysis of pretreated wheat straw

Pretreatment method	Source of enzyme used for saccharification	Temperature (°C)	Enzyme loading (per g of wheat straw)	Reducing sugars (mg/*g* wheat straw)	Reference
Acetic acid/steam explosion/supercritical carbon dioxide pretreatment	Commercial cellulase (ASA Spezialenzyme GmbH, Germany)	50	15 FPU	336.41	[30]
Hydrothermal pretreatment	Cellic CTec2 (Sigma-Aldrich, Germany)	50	100 CMCase	480.00	[31]
Ammonium sulfite pretreatment with sodium carbonate supplementation	Commercial cellulase and xylanase (Imperial Jade Bio-Technology Co., Ltd. Yinchuan, China)	50	35 FPU cellulase and 70 FXU xylanase	413.00	[53]
[EMIM][Ac] Ionic liquid pretreatment	*Sporotrichum thermophile* xylanase	40	160 U	281.00	[33]
Acid pretreatment	*T. reesei* NCIM 1186	50	30.85 CMCase	371.44	[54]
Alkali pretreatment	*Phoma exigua* secretome	55	15.23 FPU	101.16	[34]
Acid pretreatment	*Penicillium janthinellum* EMS-UV-8 cellulase	50	20 FPU	376.00	[55]
Alkali pretreatment	*Aspergillus fumigatus*	50	30 FPU	675.36	Present study

## Conclusions

The present study evaluated the use of an unconventional but abundant and recalcitrant lignocellulosic agro-forest residue, i.e., pine sawdust (PSD), for the production of the hydrolytic enzymes using *A. fumigatus*, isolated from an ancient Złoty Stok gold mine. The findings demonstrated that *A. fumigatus* has a significant potential for producing high titers of cellulases and hemicellulases at low cost under SSF. The statistical model optimization (RSM) endorsed that enzyme production primarily affected by physical characteristics like pH, temperature, and time, whose optimal selection increased the yield twofold. The secretome profiling supported the lignocellulosic biomass valorization potential due to the repertoire of diverse CAZymes. Lastly, the high reducing sugar yields obtained from saccharification of pretreated agro-forest residues indicated the potential of this low-cost bioprocess for valorizing waste streams with concomitant generation of high-value commercial commodities with diverse applications.

## Materials and methods

### Isolation, screening, and identification of potential lignocellulolytic fungus

The samples were collected from an ancient gold mine (Złoty Stok) in southwest Poland (area descriptions and parameters) mentioned by [16]. All samples were transferred to the laboratory in sterile falcons and plastic bags and stored at 4 ℃. To evaluate their cellulolytic potential, a substrate-specific enrichment approach was used. For the isolation process, diluted samples were mixed with Reese’s minimal medium (RMM) with the following composition (g/L): 0.5 Peptone (Protease) (CAS number: 91079-38-8); 2.0 KH_2_PO4; 0.3 MgSO_4_; 0.3 KNO_3_; 1.4 (NH_4_)_2_SO_4_; 0.3 CaCl_2_; including micronutrients (mg/L), i.e., 5 mg FeSO_4_; 1.4 mg ZnSO_4_; 2 mg CoCl_2_; 5 mg MnSO_4_, pH 6.0 supplemented with 1% carboxymethyl cellulose (CMC). The inoculated flask with samples was incubated at 25 ℃ for 7 days. Following enrichment, isolates were grown on RMM with 1% CMC (*w*/*v*) as a substrate using the spread plate technique and producing isolates were selected based on plate assay [37], purified, and maintained on potato dextrose agar (PDA) plates. The fungal cultures were stored at 4 ℃ and subcultured at monthly intervals. The screened sample identified by rDNA sequences was amplified using universal primer pairs, pITS-1 (5-TCCGTAGGTGAACCTGCGG-3) and pITS-4 (5-TCCTCCGCTTATTGATATGC-3). The DNA from the amplified fragments was sequenced at Eurofins Polska (https://www.eurofins.pl/), and the sequenced amplified product was then BLAST searched at the NCBI database. The MEGA 11 (molecular evolutionary genetics analysis) tool created a phylogenetic tree using the neighbor-joining method to estimate evolutionary distances [38]. Based on quantitative and qualitative screening for higher cellulolytic activity among all isolated fungus strains (data not shown), *Aspergillus fumigatus* ZS_AF (NCBI accession number OM258166) was chosen for further study.

### Biomass feedstock and chemicals

PSD and WS were sourced locally from the Poland countryside, partially chopped, and sieved with 20–40 mesh size. Both substrates were dried and stored at room temperature (~22 ℃) for future studies. The chemical composition of both untreated PSD and WS (% *w*/*w*) were determined by standard NREL procedures as 44.8 ± 0.2; 32 ± 0.25 cellulose, 27.9 ± 0.4; 45.9 ± 0.3 hemicelluloses, and 23.9 ± 0.4; 14.2 ± 0.5 lignin, respectively [39]. All analytical grade chemicals are purchased from Sigma Aldrich (St. Louis, MO, USA).

### Solid-state fermentation for hydrolytic enzyme production utilizing PSD as substrate

Five grams of coarsely crushed and sieved (1 mm mesh size) PSD with a substrate-to-moisture ratio of 1:3 utilizing RMM were employed in 200 ml Erlenmeyer flasks for the SSF procedure. *A. fumigatus* spores were grown on PDA for inoculum development. The spores were harvested in sterile Tween-80 (0.1% *v*/*v*) as it makes the dispersion on water easier, yielding a more consistent procedure for inoculum preparation. Subsequently, each flask was inoculated with a concentration of ~1 × 10^8^/ ml spores and incubated under static conditions at 30 ℃ for 5 days. The extraction procedure included using a 0.05 M citrate buffer pH 6.0, followed by 1 h of constant shaking at 150 rpm at 30 ℃. The resulting crude extract was subsequently passed through two layers of muslin cloth to remove any remaining solids, and it was then centrifuged at 10,000 rpm for 20 min at 4 ℃. The clear supernatant obtained after centrifugation was stored at 4 ℃ and used for additional investigations of the activities of several hydrolytic enzymes.

### Statistical approach for optimizing hydrolytic enzyme production

In the present study, the effect of physical factors such as media pH range (5.0–11.0), temperature (15–40 ℃), and time (3–9 days) on enzyme production was carried out by the COVT approach. The output of *A. fumigatus* enzymes was significantly affected by these parameters. To further boost the enzyme production, these three parameters were optimized using RSM, including rotatable central composite design (RCCD) using Design-Expert version 7.0.0 software (Stat-Ease Corporation, USA). This software generated 24-factorial design at five levels (−α, −1, 0, +1, +α). Table 1 displays the design matrix comprising 20 experimental runs. The matrix has six axial points, six center points, and eight random factorial points. The response for the designed model was collected from several enzyme activities (i.e., CMCase, xylanase, β-glucosidases, and FPase). The RSM experiments' data were evaluated using analysis of variance (ANOVA). This enabled the study of regression coefficients, prediction equations, and case statistics. The experimental outcomes of RSM were fitted with a second-order polynomial equation (Eq. 1) to enable the estimation of the response variable using independent variables:

1 $$Y={\beta }_{0}+{\sum }_{j=1}^{k}{\beta }_{j}{X}_{j}+ {\sum }_{j=1}^{k}{\beta }_{jj}{X}_{j}^{2}+ {\sum }_{i}{\sum }_{<j=2}^{k}{\beta }_{ij}{X}_{i}{X}_{j}+ {e}_{i}.$$

Y represents the response variable in the equation. The intercept coefficient for the model is *β*_0_. The interaction between the linear, quadratic, and second-order terms are denoted by the coefficients *β*_*j*_*, β*_*jj*_*, and β*_*ij*_*,* respectively. The independent variables are represented by *X*_*i*_ and *X*_*j*_*,* where *i* and *j* are values ranging from 1 to k. This study had three independent parameters (*k* = 3). The error is represented by *e*_*i*_. The statistical model was verified to reflect all variables in the design space accurately. To illustrate how significant variables affected the response, three-dimensional graphics were created.

### Assays for determining enzymatic activities

The crude enzyme produced from *A. fumigatus* utilizing PSD during the SSF process was tested for endoglucanase (CMCase), xylanase, β-glucosidase (cellobiase), and filter paper activity (FPase). The endoglucanase activity was examined by dissolving 2% CMC (*w*/*v*) in 0.05 M citrate buffer pH 6.0 and incubating at 50 ℃ for 30 min. The dinitrosalicylic acid (DNS) method determined the total reducing sugars by UV–visible spectrophotometer at 540 nm. For exoglucanase activity, 50 mg Whatman Filter paper No. 1 was mixed in 0.05 M citrate buffer (pH 6.0) and incubated at 50 ℃ for 60 min to determine the reducing sugar using the Ghose [40] standard method. Similarly, 1% beechwood xylan was employed to test xylanase activity by diluting in 0.05 M citrate buffer pH 6.0 and incubating at 50 ℃ for 30 min. One unit (U) of xylanase activity is the amount of enzyme that releases 1 µmol of xylose per minute. The activity of β-glucosidase was evaluated using p-nitrophenyl-α-D-glucopyranoside as a substrate, and the reaction was stopped by adding glycine buffer (pH 10.8) after incubation. The total amount of p-nitrophenol was determined by measuring absorbance at 405 nm. [41].

### Secretome analysis and protein identification by LC–MS/MS

*A. fumigatus* enzyme extracted in supernatant form during the SSF process was further used for secretome analysis. The proteins were precipitated overnight in ice-cold acetone (80%), and their concentrations were determined using the Bradford method [42]. The mixture was centrifuged for 15 min, and the resultant air-dried pellet was resuspended in 0.2 M ammonium bicarbonate. The protein (20 µg) was subsequently loaded onto 12% SDS-PAGE gel, and the bands were visualized using Coomassie brilliant blue G-250. The gel is chopped into smaller pieces after being sliced into three or four sections. The diced gel was rinsed and destained with 75% acetonitrile (ACN) solution and 25 mM triethylammonium bicarbonate buffer (TEAB). Furthermore, the reduction and alkylation reactions were carried out with 10 mM dithiothreitol and 50 mM iodoacetamide, respectively. The gel pieces were washed twice with TEAB and dehydrated using 100% ACN to remove access to reducing and alkylating agents. The fragmented gel was processed overnight with trypsin at 37 ℃; the resultant tryptic digest was extracted with 5% acetonitrile and 0.1% formic acid before being subjected to LC–MS/MS analysis [21, 23]. Protein identification was carried out using Mascot 2.4.01, and the resulting protein entry was systematically matched against the UniProt database for *A. fumigatus* [43]. The identified proteins were categorized into different glycoside hydrolases (GH) families based on the dbCAN2 tool [44].

### Pretreatment and structural analysis of agro-forest residues subjected to saccharification by produced hydrolytic enzymes

Following Jin et al. [35] with some adjustments, both PSD and WS were treated with 5% (*w*/*v*) alkali (NaOH) solution. The suspension was kept in an oil bath at 120 ℃ for 30 min. After cooling, the pretreated solids were meticulously washed with distilled water until they attained a neutral pH. The solid leftovers were collected and dried overnight. Similarly, the structural change on raw, alkali-pretreated, hydrolyzed PSD and WS was conducted using scanning electron microscopy (SEM). Fourier transform infrared spectroscopy (FTIR) for observing changes in the functional groups and X-ray diffraction (XRD) to elucidate the cellulose crystallinity by determining the crystallinity index (CrI) was calculated using Eq. 2 [45]:

2 $$ {\text{Crystallinity index }}\left( {{\text{CrI}}} \right) \, = \frac{{{\text{Crystalline portion }}\left( {{\text{I}}_{{00{2}}} } \right) \, - {\text{ amorphous portion }}\left( {{\text{I}}_{{{\text{AM}}}} } \right)}}{{\text{Crystalline portion}}} \times 100\% , $$

where (I_002_ = intensity at 2θ = 22.5 and I_AM_ = intensity at 2θ = 18.5).

### Enzymatic hydrolysis of lignocellulosic substrates

The enzyme attained from the SSF process under optimum conditions was utilized to hydrolyze both substrates, i.e., PSD and WS. Specifically, 150 FPU/gds of PSD and 30 FPU/gds of WS containing 0.005% (*w*/*v*) sodium azide were used to saccharify the substrates at 50 ℃ under 150 rpm for durations of 24, 48, and 72 h. Untreated PSD and WS were used as controls, with the same enzyme concentrations as the treated samples. After centrifugation, the clear supernatant was collected at various time intervals (24, 48, and 72 h), and the reducing sugars were measured using the DNS method [46]. Each condition was tested in triplicate. The hydrolysis yield was determined using Eq. 3 [47]:

3 $$ {\text{Yield of hydrolysis }}\left( \% \right)\frac{{{\text{Reducing sugar }}\left( {\text{g}} \right){\text{ x }}0.{9}}}{{{\text{Polysaccharide in a substrate }}\left( {\text{g}} \right)}} \times 100. $$

### Statistical analysis

RSM was used to investigate the optimization data subjected to analysis of variance (ANOVA) in a rotatable central composite design (RCCD). The experiment values shown in graphs and tables are the mean±SD of three replicates determined in MS Excel.

### Supplementary Information

**Additional file 1: Fig. S1 a** A phylogenetic tree for *A. fumigatus* based on ITS sequence, **b** *A. fumigatus* spores grown over PDA plates; magnification captured at 4000× **c** *A. fumigatus* growth over PSD, magnification captured at 4000×. **Fig. S2** Residual diagnostics of contour surface of the quadratic model by Normal plot of residuals for **a** CMCase, **b** xylanase, **c** β-glucosidase and **d** FPase production. **Fig. S3** FTIR spectra of untreated, alkali-pretreated, and hydrolyzed **a** Pine sawdust (PSD) and; **b** Wheat straw (WS). **Fig. S4** XRD diffraction of untreated, alkali-pretreated and hydrolyzed **a** Pine sawdust (PSD) and **b** Wheat straw (WS). **Table S1** Regression analysis for the production of CMCase enzyme by *A. fumigatus* under SSF for quadratic response surface model fitting ANOVA. Where X1= pH, X2= Temperature and X3= Time. **Table S2** Regression analysis for the production of xylanase enzyme by *A. fumigatus* under SSF for quadratic response surface model fitting ANOVA. Where X1= pH, X2= Temperature and X3= Time. **Table S3 **Regression analysis for the production of β-glucosidase enzyme by *A. fumigatus* under SSF for quadratic response surface model fitting ANOVA. Where X1= pH, X2= Temperature and X3= Time. **Table S4 **Regression analysis for the production of FPase enzyme by *A. fumigatus* under SSF for quadratic response surface model fitting ANOVA. Where X1= pH, X2= Temperature and X3= Time.

## Data Availability

All data generated or analyzed during this study are included in this article.

## Abbreviations

PSD: Pine sawdust
RSM: Response surface methodology
CAZymes: Carbohydrate-active enzymes
SSF: Solid-state fermentation
CMC: Carboxymethyl cellulose
RMM: Reese’s minimal medium
WS: Wheat straw
PDA: Potato dextrose agar
RCCD: Rotatable central composite design
ANOVA: Analysis of variance
DNS: Dinitrosalicylic acid
ACN: Acetonitrile
TEAB: Triethylammonium bicarbonate buffer
GH: Glycoside hydrolases
CE: Carbohydrate esterases
CBM: Carbohydrate-binding module
COVT: Constant one variable at a time
SEM: Scanning electron microscopy
XRD: X-ray diffraction
CrI: Crystallinity index
FTIR: Fourier transform infrared spectroscopy
FPase: Filter paper activity
NREL: National renewable energy laboratory
