# Supplementary material for: Bioprospecting CAZymes repertoire of Aspergillus fumigatus for eco-friendly value-added transformations of agro-forest biomass
Source: Biotechnol Biofuels Bioprod. 2024 Jan 3;17:3. doi: 10.1186/s13068-023-02453-6 (PMC10765743; doi:10.1186/s13068-023-02453-6)
Supplement: Supplementary file 1 — Additional file 1: Fig. S1 a A phylogenetic tree for A. fumigatus based on ITS sequence, b A. fumigatus spores grown over PDA plates; magnification captured at 4000× c A. fumigatus growth over PSD, magnification captured at 4000×. Fig. S2 Residual diagnostics of contour surface of the quadratic model by Normal plot of residuals for a CMCase, b xylanase, c β-glucosidase and d FPase production. Fig. S3 FTIR spectra of untreated, alkali-pretreated, and hydrolyzed a Pine sawdust (PSD) and; b Wheat straw (WS). Fig. S4 XRD diffraction of untreated, alkali-pretreated and hydrolyzed a Pine sawdust (PSD) and b Wheat straw (WS). Table S1 Regression analysis for the production of CMCase enzyme by A. fumigatus under SSF for quadratic response surface model fitting ANOVA. Where X1= pH, X2= Temperature and X3= Time. Table S2 Regression analysis for the production of xylanase enzyme by A. fumigatus under SSF for quadratic response surface model fitting ANOVA. Where X1= pH, X2= Temperature and X3= Time. Table S3 Regression analysis for the production of β-glucosidase enzyme by A. fumigatus under SSF for quadratic response surface model fitting ANOVA. Where X1= pH, X2= Temperature and X3= Time. Table S4 Regression analysis for the production of FPase enzyme by A. fumigatus under SSF for quadratic response surface model fitting ANOVA. Where X1= pH, X2= Temperature and X3= Time. [file 13068_2023_2453_MOESM1_ESM.docx]

**Supplementary materials**

**
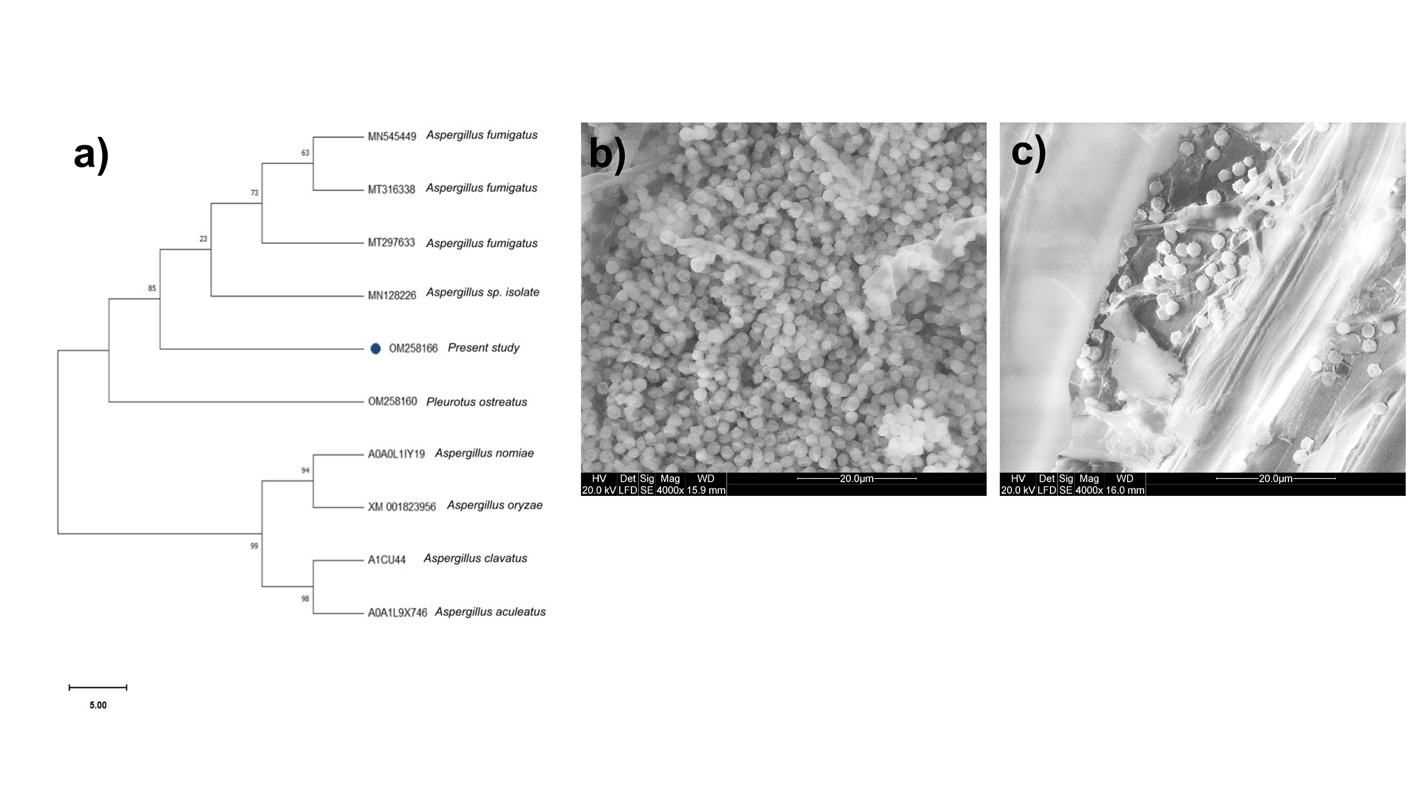
**

**Fig. S1. a)** A phylogenetic tree for *A. fumigatus* based on ITS sequence, **b)** *A. fumigatus* spores grown over PDA plates; magnification captured at 4000x **c)** *A. fumigatus* growth over PSD, magnification captured at 4000x.


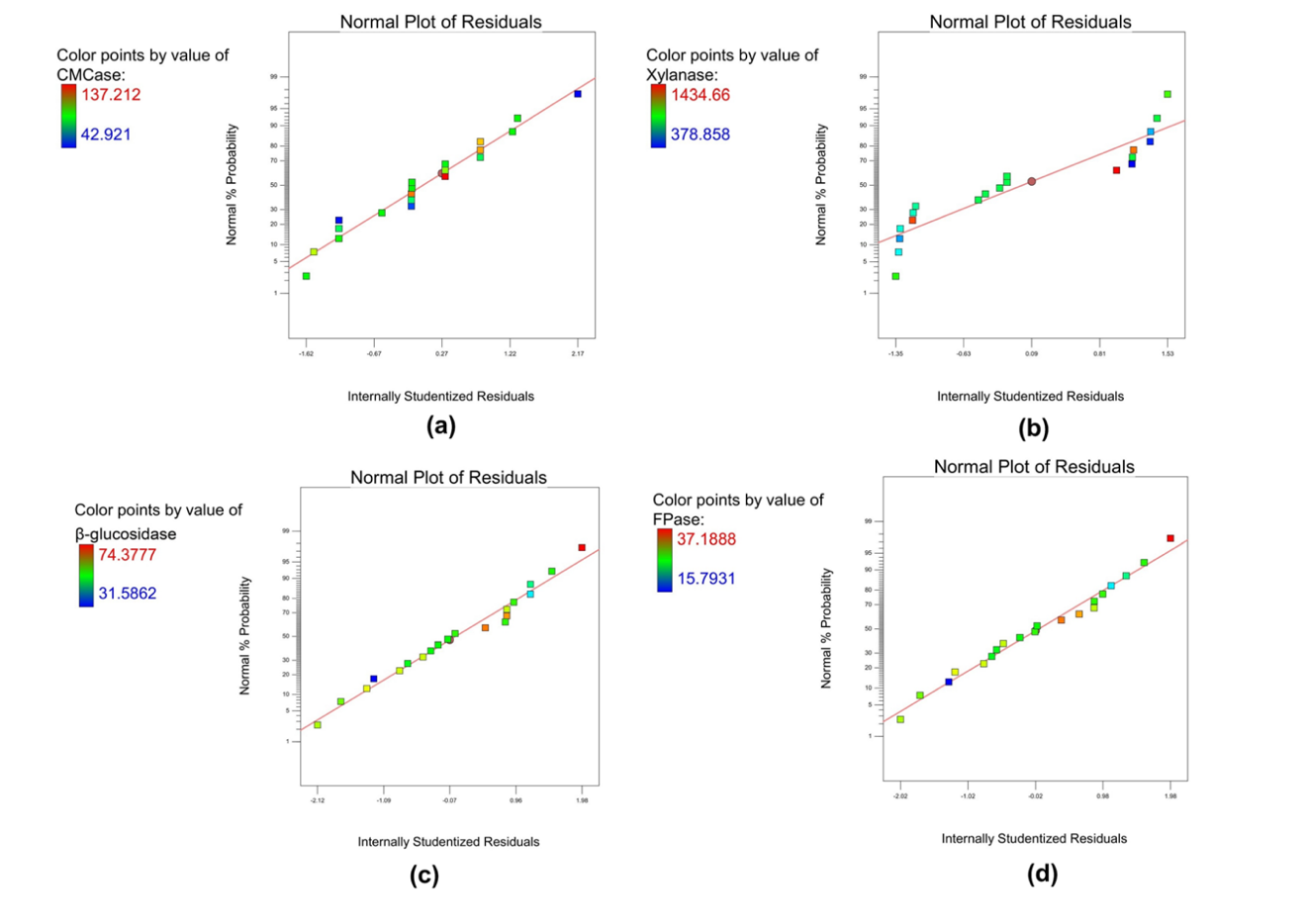


**Fig. S2.** Residual diagnostics of contour surface of the quadratic model by Normal plot of residuals for **(a)** CMCase, **(b)** xylanase, **(c)** β-glucosidase and **(d)** FPase production.


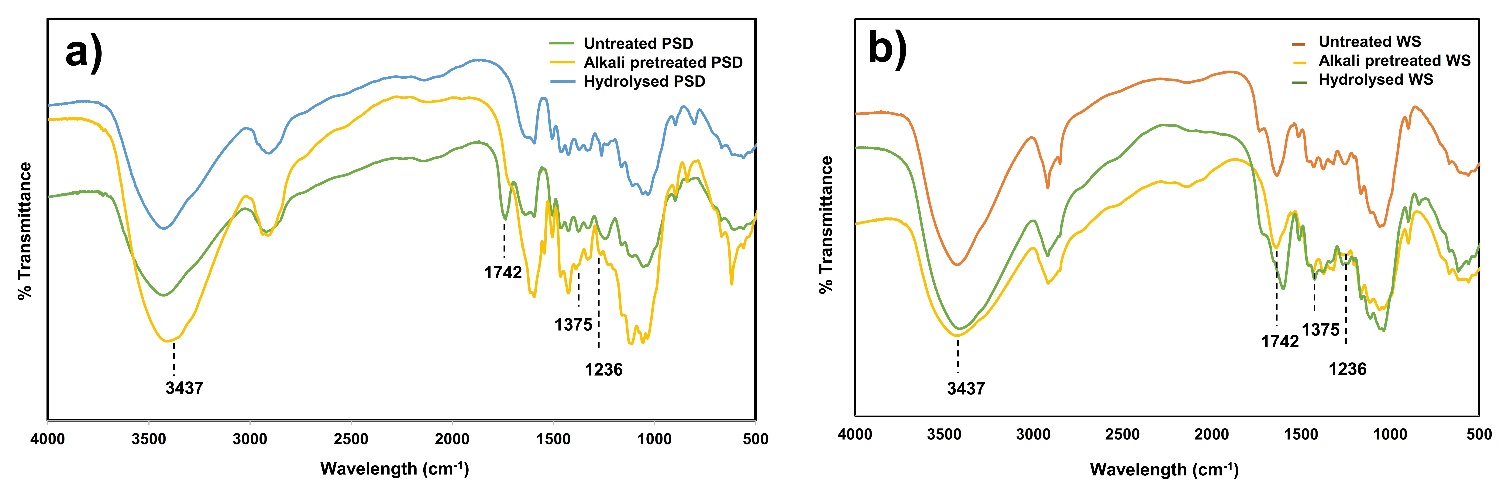


**Fig. S3**. FTIR spectra of untreated, alkali-pretreated, and hydrolyzed **a)** Pine sawdust (PSD) and; **b)** Wheat straw (WS).


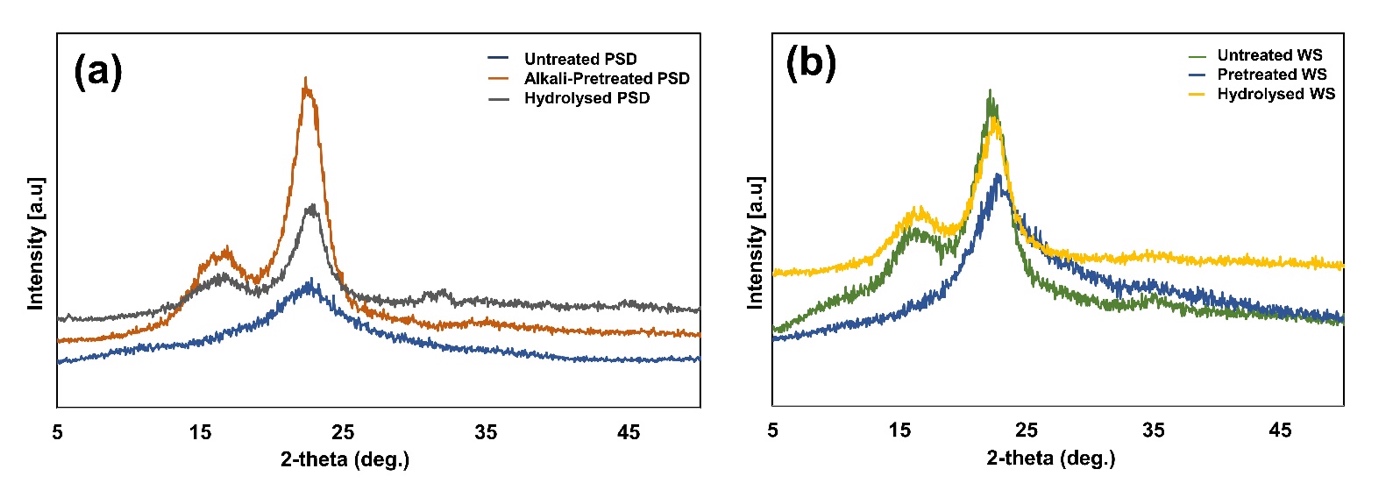


**Fig. S4.** XRD diffraction of untreated, alkali-pretreated and hydrolyzed **a)** Pine sawdust (PSD) and **b)** Wheat straw (WS).

**Table S1** Regression analysis for the production of CMCase enzyme by *A. fumigatus* under SSF for quadratic response surface model fitting ANOVA. Where X1= pH, X2= Temperature and X3= Time.

| **Source** | **Sum of square** | **Degree of freedom** | **Standard error** | **Mean square** | ***F* Value** | **p-value (Prob > F)** |  |
| --- | --- | --- | --- | --- | --- | --- | --- |
| Model | 11947.38 | 9 | 0.14 | 1327.49 | 11390.12 | <0.0001 | Significant |
| X1 | 7.28 | 1 | 0.092 | 7.27 | 62.44 | <0.0001 |  |
| X2 | 10291.14 | 1 | 0.092 | 10291.14 | 88300.12 | <0.0001 |  |
| X3 | 502.06 | 1 | 0.092 | 502.06 | 4307.81 | <0.0001 |  |
| X12 | 74.58 | 1 | 0.12 | 74.58 | 639.88 | <0.0001 |  |
| X13 | 17.65 | 1 | 0.12 | 17.65 | 151.41 | <0.0001 |  |
| X23 | 1043.23 | 1 | 0.12 | 1043.23 | 8951.12 | <0.0001 |  |
| X_1_^2^ | 0.089 | 1 | 0.090 | 0.089 | 0.76 | 0.4036 |  |
| X_2_^2^ | 1.90 | 1 | 0.090 | 1.90 | 16.31 | 0.0024 |  |
| X_3_^2^ | 10.30 | 1 | 0.090 | 10.30 | 88.42 | <0.0001 |  |
| Residual | 1.17 | 10 |  | 0.12 |  |  |  |
| Lack of fit | 0.55 | 5 |  | 0.11 | 0.89 | 0.5491 | Not significant |
|  |  |  |  |  |  |  |  |
| Std. Dev. |  | 0.34 |  |  |  |  |  |
| Mean |  | 90.83 |  |  |  |  |  |
| C.V.% |  | 0.38 |  |  |  |  |  |
| Ade. Precision | | 392.084 |  |  |  |  |  |
| R^2^ |  | 0.9999 |  |  |  |  |  |
| Adjusted R^2^ | | 0.9998 |  |  |  |  |  |
| Predicted R^2^ | | 0.9995 |  |  |  |  |  |

**Table S2** Regression analysis for the production of xylanase enzyme by *A. fumigatus* under SSF for quadratic response surface model fitting ANOVA. Where X1= pH, X2= Temperature and X3= Time.

| **Source** | **Sum of square** | **Degree of freedom** | **Standard error** | **Mean square** | ***F* Value** | **p value (Prob > F)** |  |
| --- | --- | --- | --- | --- | --- | --- | --- |
| Model | 1.516E+006 | 9 | 3.67 | 1.684E+005 | 2082.73 | <0.0001 | Significant |
| X1 | 7.973E+005 | 1 | 2.43 | 7.973E+005 | 9859.94 | <0.0001 |  |
| X2 | 1.100E+005 | 1 | 2.43 | 1.100E+005 | 1359.88 | <0.0001 |  |
| X3 | 475.14 | 1 | 2.43 | 475.14 | 5.88 | 0.0358 |  |
| X12 | 3.560E+005 | 1 | 3.18 | 3.560E+005 | 4403.13 | <0.0001 |  |
| X13 | 1921.10 | 1 | 3.18 | 1921.10 | 23.76 | 0.0006 |  |
| X23 | 39025.89 | 1 | 3.18 | 39025.89 | 482.63 | <0.0001 |  |
| X_1_^2^ | 29330.29 | 1 | 2.37 | 29330.29 | 362.73 | <0.0001 |  |
| X_2_^2^ | 1.335E+005 | 1 | 2.37 | 1.335E+005 | 1650.76 | <0.0001 |  |
| X_3_^2^ | 27299.80 | 1 | 2.37 | 27299.80 | 337.62 | <0.0001 |  |
| Residual | 808.60 | 10 |  | 80.86 |  |  |  |
| Lack of fit | 639.18 | 5 |  | 127.84 | 3.77 | 0.0857 | Not significant |
|  |  |  |  |  |  |  |  |
| Std. Dev. |  | 8.99 |  |  |  |  |  |
| Mean |  | 829.39 |  |  |  |  |  |
| C.V.% |  | 1.08 |  |  |  |  |  |
| Ade. Precision | | 166.179 |  |  |  |  |  |
| R^2^ |  | 0.9995 |  |  |  |  |  |
| Adjusted R^2^ | | 0.9990 |  |  |  |  |  |
| Predicted R^2^ | | 0.9966 |  |  |  |  |  |

**Table S3** Regression analysis for the production of β-glucosidase enzyme by *A. fumigatus* under SSF for quadratic response surface model fitting ANOVA. Where X1= pH, X2= Temperature and X3= Time.

| **Source** | **Sum of square** | **Degree of freedom** | **Standard error** | **Mean square** | ***F* Value** | **p value (Prob > F)** |  |
| --- | --- | --- | --- | --- | --- | --- | --- |
| Model | 1706.30 | 9 | 0.78 | 189.59 | 52.29 | <0.0001 | Significant |
| X1 | 0.58 | 1 | 0.52 | 0.58 | 0.16 | 0.6978 |  |
| X2 | 1195.71 | 1 | 0.52 | 1195.71 | 329.81 | <0.0001 |  |
| X3 | 10.70 | 1 | 0.52 | 10.70 | 2.95 | 0.1166 |  |
| X12 | 41.98 | 1 | 0.67 | 41.98 | 11.58 | 0.0067 |  |
| X13 | 5.18 | 1 | 0.67 | 5.18 | 1.43 | 0.2596 |  |
| X23 | 144.70 | 1 | 0.67 | 144.70 | 39.91 | <0.0001 |  |
| X_1_^2^ | 148.06 | 1 | 0.50 | 148.06 | 40.84 | <0.0001 |  |
| X_2_^2^ | 37.86 | 1 | 0.50 | 37.86 | 10.44 | 0.0090 |  |
| X_3_^2^ | 114.11 | 1 | 0.50 | 114.11 | 31.47 | 0.0002 |  |
| Residual | 36.25 | 10 |  | 3.63 |  |  |  |
| Lack of fit | 29.65 | 5 |  | 5.93 | 4.49 | 0.0624 | Not significant |
|  |  |  |  |  |  |  |  |
| Std. Dev. |  | 1.9 |  |  |  |  |  |
| Mean |  | 56.39 |  |  |  |  |  |
| C.V.% |  | 3.38 |  |  |  |  |  |
| Ade. Precision | | 29.068 |  |  |  |  |  |
| R^2^ |  | 0.9792 |  |  |  |  |  |
| Adjusted R^2^ | | 0.9605 |  |  |  |  |  |
| Predicted R^2^ | | 0.8648 |  |  |  |  |  |

**Table S4** Regression analysis for the production of FPase enzyme by *A. fumigatus* under SSF for quadratic response surface model fitting ANOVA. Where X1= pH, X2= Temperature and X3= Time.

| **Source** | **Sum of square** | **Degree of freedom** | **Standard error** | **Mean square** | ***F* Value** | **p value (Prob > F)** |  |
| --- | --- | --- | --- | --- | --- | --- | --- |
| Model | 421.01 | 9 | 0.41 | 46.78 | 47.27 | <0.0001 | Significant |
| X1 | 0.20 | 1 | 0.27 | 0.20 | 0.20 | 0.6641 |  |
| X2 | 290.21 | 1 | 0.27 | 290.21 | 293.27 | <0.0001 |  |
| X3 | 2.49 | 1 | 0.27 | 2.49 | 2.52 | 0.1435 |  |
| X12 | 9.47 | 1 | 0.35 | 9.47 | 9.57 | 0.0114 |  |
| X13 | 1.38 | 1 | 0.35 | 1.38 | 1.39 | 0.2657 |  |
| X23 | 39.46 | 1 | 0.35 | 39.46 | 39.87 | <0.0001 |  |
| X_1_^2^ | 37.05 | 1 | 0.26 | 37.05 | 37.44 | 0.0001 |  |
| X_2_^2^ | 9.16 | 1 | 0.26 | 9.16 | 9.26 | 0.0124 |  |
| X_3_^2^ | 29.90 | 1 | 0.26 | 29.90 | 30.21 | 0.0003 |  |
| Residual | 9.90 | 10 |  | 0.99 | 3.75 |  |  |
| Lack of fit | 7.81 | 5 |  | 1.56 |  | 0.0867 | Not significant |
|  |  |  |  |  |  |  |  |
| Std. Dev. |  | 0.99 |  |  |  |  |  |
| Mean |  | 28.15 |  |  |  |  |  |
| C.V.% |  | 3.53 |  |  |  |  |  |
| Ade. Precision | | 27.653 |  |  |  |  |  |
| R^2^ |  | 0.9770 |  |  |  |  |  |
| Adjusted R^2^ | | 0.8540 |  |  |  |  |  |
| Predicted R^2^ | | 0.9966 |  |  |  |  |  |
